# Chronic treatment with serelaxin mitigates adverse remodeling in a murine model of ischemic heart failure and modulates bioactive sphingolipid signaling

**DOI:** 10.1038/s41598-022-12930-x

**Published:** 2022-05-25

**Authors:** Teja Devarakonda, Juan Valle Raleigh, Adolfo G. Mauro, Johana M. Lambert, Lauren Ashley Cowart, Fadi N. Salloum

**Affiliations:** 1 Division of Cardiology, Department of Internal Medicine, Pauley Heart Center, Virginia Commonwealth University, 1101 East Marshall Street, Room 7-070, Box 980204, Richmond, VA 23298 USA; 2 Department of Physiology and Biophysics, Virginia Commonwealth University, Richmond, VA USA; 3 Department of Biochemistry and Molecular Biology, Virginia Commonwealth University, Richmond, VA USA; 4 Hunter Holmes McGuire Veterans’ Affairs Medical Center, Richmond, VA USA

**Keywords:** Cardiovascular diseases, Biochemistry, Lipidomics

## Abstract

Relaxin is a pleiotropic hormone demonstrated to confer cardioprotection in animal models of myocardial infarction and ischemic heart failure by modulating inflammation, fibrosis and arrhythmogenesis. Several of these pathways in the ischemic myocardium are intricately tied with the downstream signaling of bioactive sphingolipids, which play an active role during post-infarction remodeling. In this current study, we examined the effects of relaxin on sphingosine 1-phosphate (S1P), and the potential benefits of relaxin treatment on cardiac health in a rodent model of ischemic heart failure. Acute (30 min) and sub-acute (24 h) treatment of primary cardiomyocytes with serelaxin (recombinant human relaxin-2) increased the cardiomyocyte content of S1P. In the rodent model, treatment with relaxin for 28 days following myocardial ischemia by way of permanent left coronary artery occlusion improved survival and cardiac function, reduced fibrosis and apoptosis, and mitigated the expression of several pro-inflammatory and pro-fibrotic markers. The expression of beclin-1 (autophagy marker) was also reduced. The expression of S1P was significantly higher in cardiac tissue and plasma samples extracted from serelaxin-treated mice at day 28. In conclusion, our studies show a significant protection from relaxin in ischemic heart disease, and demonstrate the association between relaxin signaling and S1P generation.

## Introduction

Sphingolipids and associated metabolites are increasingly associated with regulation of several pathways involving cellular survival, senescence, metabolism, and protein quality control^1,2^. Among the various bioactive sphingolipids, sphingosine-1-phosphate (S1P) is implicated in several homeostatic pathways within the mammalian cardiovascular system. Sphingosine is derived from ceramide, which in turn is produced by the de novo synthesis pathway involving serine and palmitoyl CoA, or through breakdown of the phospholipid sphingomyelin^3^. The phosphorylation of sphingosine, which regulates the bioactive pool of S1P, is mediated by the enzymes sphingosine kinases 1 & 2 (SphK1 & 2). While SphK1 maintains a predominantly cytoplasmic presence, SphK2 possesses a nuclear localization sequence (NLS) and a nuclear export signal (NES)^1^. Therefore, S1P-mediated effects are highly compartmentalized within cellular organelles^1,4^. S1P exerts its downstream effects by activating the S1P receptors 1–5 (S1PRs). S1PRs are a subset of GPCRs, and signal transduction upon activation occurs through the mobilization of recruited G proteins. Of the 5 receptor family members, S1PR1, 2 & 3 are predominantly expressed in cardiac cells^1,2,5^. In cardiomyocytes, S1PR1 & 3 signaling is recognized to confer protection in the context of ischemia–reperfusion (IR) injury. S1PR1 activation leads to phosphorylation of Akt and Erk 1/2, and activation of PKCε. These targets play a major role in transducing preconditioning-associated protective mechanisms in the heart during IR injury^1,6^. Downregulation of S1PR1 activity in in vitro models of stress-induced cardiomyopathy led to increases in cardiac autophagy, adverse cardiac function and pathologic hypertrophy^7^. Nevertheless, contrasting reports indicate a detrimental role of S1P signaling in the remodeling heart post ischemia, as increased activity of SphK1/S1P/S1PR1 axis was attributed to cause pro-inflammatory responses weeks following myocardial infarction (MI)^8^. Therefore, the precise mechanisms involved in S1P induced cardiac effects—during early stages of reperfusion and over the course of adverse remodelling—are yet to be elucidated.

Relaxin is a pleiotropic mammalian hormone shown to induce various protective effects in the ischemic myocardium, including reduced inflammasome activity^9^ and fibrosis^10^, and mitigation of arrhythmogenesis^11,12^. The intersection of relaxin signaling and S1P-mediated mechanisms is of recent interest, as relaxin has been shown to increase SphK activity, and consequently, S1P production in primary neonatal cardiomyocytes^13^. The induction of S1P response was shown to be crucial in mediating the anti-fibrotic effects elicited by relaxin signaling^13^. More recently, long term (2 weeks) treatment of rats with serelaxin (recombinant human relaxin-2) led to significant changes in the myocyte lipidome, including elevated levels of precursors involved in sphingosine (ceramide and sphingomyelin) synthesis^14^. Taking these findings into consideration, we attempted to understand the role of relaxin-induced changes in sphingolipid metabolism in a permanent ligation model of ischemic heart failure, where surviving myocytes are prone to significant mechanical and metabolic stresses. We also investigated whether chronic treatment with relaxin affects myocyte autophagy in the viable myocardium of the remodeling heart, due to the inferred link between S1PR1 activation and autophagy. Finally, we attempted to utilize PF543 (SphK1 specific inhibitor at nanomolar levels in vitro) in vivo to reduce relaxin-induced changes in S1P levels for further mechanistic analysis of these novel pathways.

## Results

### Relaxin stimulates SphK1 activity in primary adult cardiomyocytes

**Figure 1:**
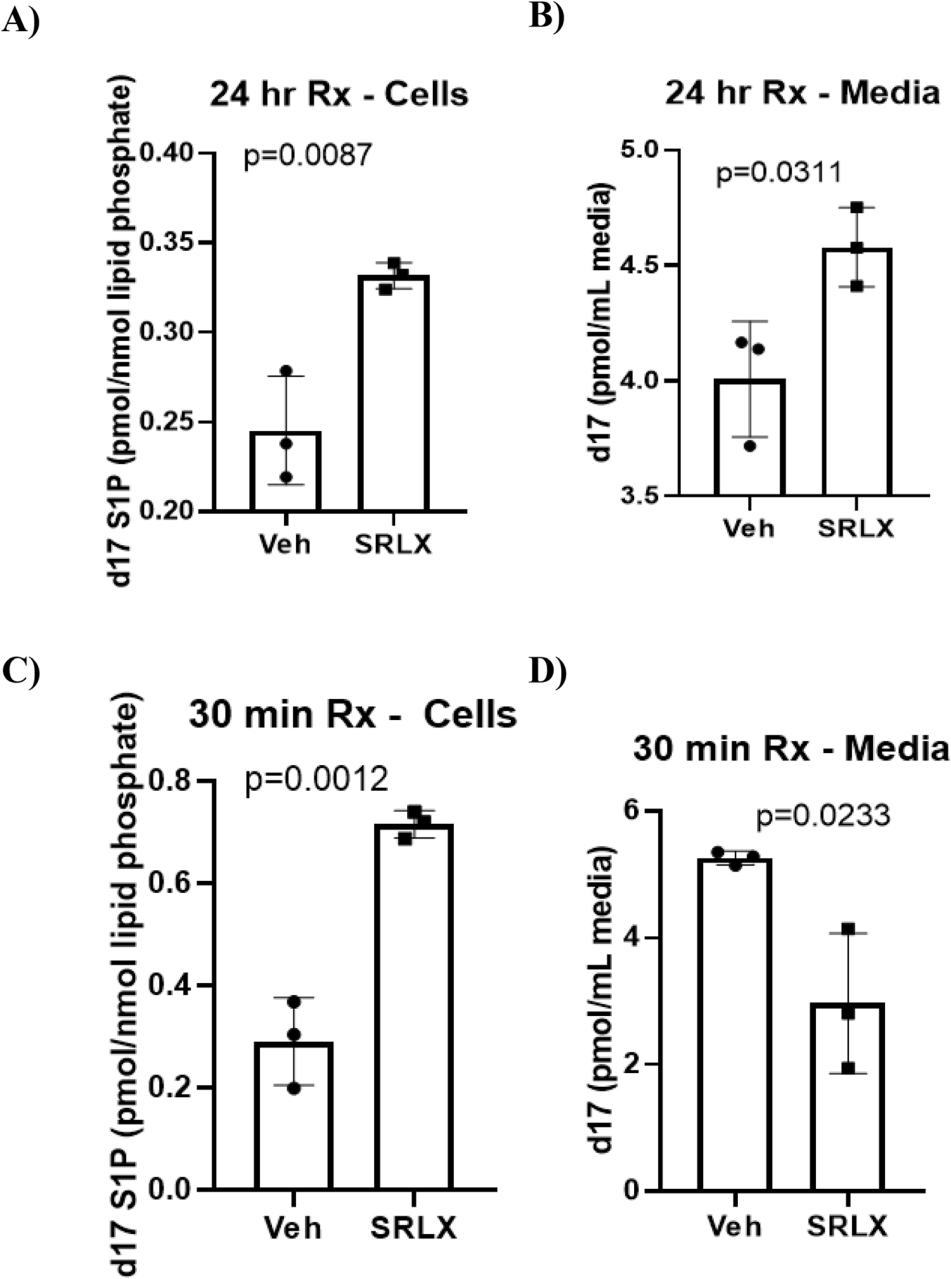
In vitro assessment of SphK1 activity in cardiomyocytes. (**A**,**B**) Media isolated from cardiomyocytes after the d17 sphingosine assay shows increased d17-S1P levels in cardiomyocytes (0.3317 ± 0.004, vs. 0.245 ± 0.017 pmol/nmol lipid phosphate in control) and media (4.57 ± 0.09 vs. 4.00 ± 0.15 pmol/ml media in control) upon treatment with serelaxin for 24 h (**C**) Co-incubation of cardiomyocytes with d17 sphingosine and serelaxin for 30 min leads to increased d17 S1P levels in the cells (0.716 ± 0.015, vs. 0.29 ± 0.049 pmol/nmol lipid phosphates in control) (**D**) In contrast to the trend in cells, the d17-S1P levels in the media after 30 min of co-incubation with serelaxin and d17 sphingosine are significantly lower (2.097 ± 0.63, vs. 5.26 ± 0.06 pmol/ml media in control). Unpaired T-test was used for all the analyses (n = 3 for each group in **A**–**D**).

In order to confirm the previously reported finding of relaxin-induced stimulation of Sphk1 in adult primary cardiomyocytes^13^, cardiac tissue was digested in a collagenase II buffer to isolate myocytes. After plating in myocyte media, cells were treated with control media or serelaxin (100 nM) infused media for 24 h (sub-acute). After the allotted time period, myocytes were co-incubated with d17 sphingosine, an unnatural and therefore traceable SphK1 substrate, for 30 min. The cell lysate and media were subsequently collected to measure d17-S1P levels via lipidomic analysis, and the results were normalized to total lipid phosphate yield per sample. Our results showed a significant increase in d17-S1P (pmol/mol of lipid phosphate) in both the cell lysate and the media (Fig. 1A,B). To compare the effects of acute treatment, freshly isolated myocytes were co-incubated with d17-sphingosine and serelaxin (100 nM) prior to lipidomic analysis. Interestingly, S1P levels were significantly elevated in the serelaxin treated cells, showing a stronger response than observed with sub-acute exposure (Fig. 1C). Paradoxically, sub-acutely treated cells had significantly lower levels of d17-S1P in the media than in the control group (Fig. 1D). Due to the strong intracellular response observed for both treatment timeframes, treatment with serelaxin likely increases SphK1 activity in cardiomyocytes.

### Relaxin protects viable myocardium in ischemic heart failure

**Figure 2:**
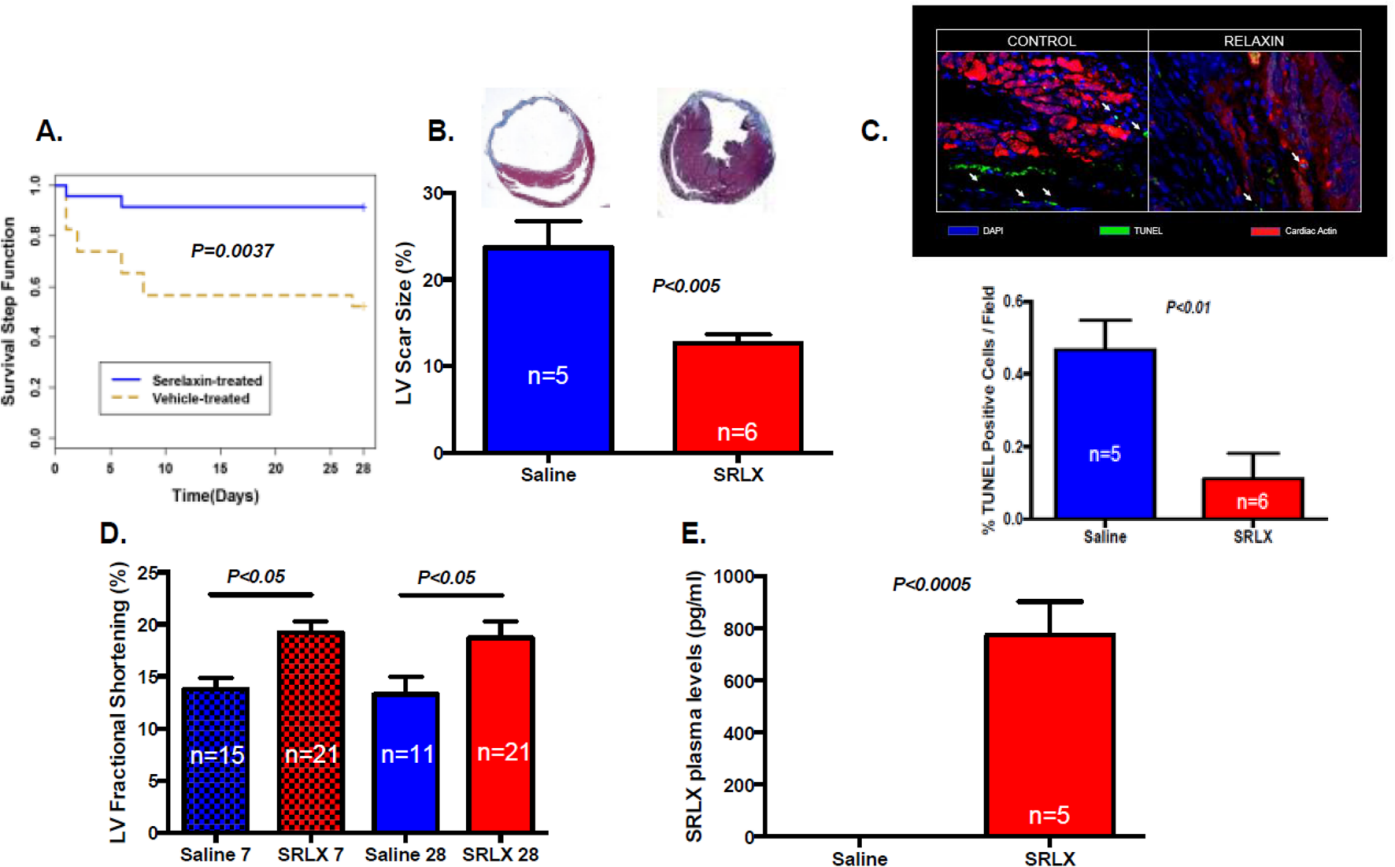
Preserved cardiac health upon chronic treatment with serelaxin. (**A**) Chronic treatment with Serelaxin (10 µg/kg/day) improves the survival in mice, as demonstrated in the Kaplan–Meier curve for survival analysis in vehicle-treated or serelaxin-treated (via osmotic mini-pumps) mice for 28 days; (**B**) The percentage of LV scar size is significantly lower in the serelaxin treated group, as assessed via trichrome staining; (**C**) The percentage of TUNEL positive cells is significantly lower in cardiac sections from serelaxin-treated mice; (**D**) Fractional shortening is significantly preserved in serelaxin-treated mice at 7 days and 28 days post induction of permanent ligation surgery; (**E**) Plasma serelaxin levels reach ~ 800 pg/ml in mice implanted with drug eluting osmotic pumps, while animals receiving saline eluting pumps have no plasma relaxin levels, as detected via ELISA.

**Figure 3:**
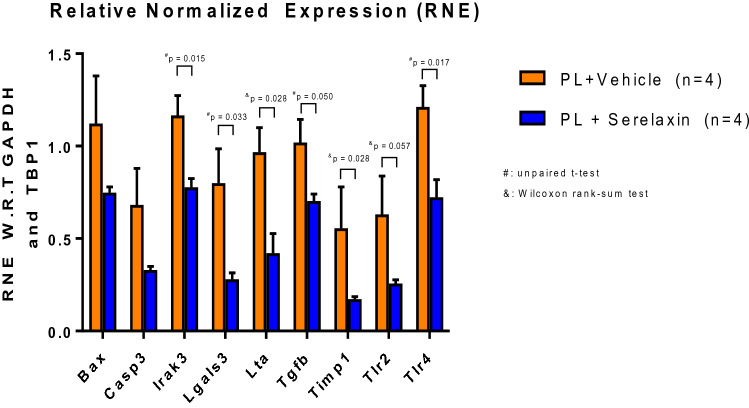
Gene expression profile upon chronic treatment with serelaxin. Expression of pro-inflammatory (Tlr2, Tlr4, Lta, Irak3), pro-apoptotic (Bax, Casp3), and profibrotic genes (Timp1, Tgfb, Lgals3) is significantly lower in animals receiving chronic serelaxin treatment. For all the data sets, unpaired T-test was utilized. However, when non-normality was detected, Wilcoxon rank-sum test was used for pair-wise comparisons. (*PL* permanent ligation, *RNE W.R.T* relative normalized expression, With respect to).

The induction of ischemic heart failure was achieved through a permanent ligation model. In this modality of myocardial injury, due to the complete lack of reperfusion of the area at risk, ischemic damage causes extensive necrosis, wall thinning and cavitary dilatation. The viable myocardium in the peri-infarct and not-at-risk (remote) regions experience ischemic stress due to increased mechanical demand, systemic neurohormonal dysregulation, and decline in metabolic efficiency—leading to further remodeling and ischemic heart failure^15^. To test the effects of long term serelaxin treatment in CD1 mice post permanent ligation-induced heart failure, mice subjected to LAD ligation were implanted with subcutaneous mini-osmotic pumps delivering either saline or serelaxin (10 µg/kg/day). Prior to implantation, mini-pumps were equilibrated in saline to allow for instantaneous delivery upon surgical placement. The Kaplan Meier curve to assess survival over the course of surgical recovery and progression toward heart failure shows significantly higher survival rates in serelaxin treated mice (Fig. 2A). Trichrome blue staining shows increased scar size (expressed as a ratio of total LV cross sectional area per section) in the saline group (Fig. 2B). The percentage of TUNEL positive cells in the viable myocardium was also higher in the saline group, indicating higher rates of apoptosis (Fig. 2C). At 7- and 28-day time points, M-mode echocardiography was performed to analyze fractional shortening (FS). FS was significantly lower in the saline-treated mice at 7- and 28-days post-surgery (Fig. 2D) Plasma samples collected from serelaxin treated mice at the time of sacrifice (28 days) had significantly higher concentrations of relaxin (Fig. 2E), validating the bioavailability of serelaxin and the feasibility of using osmotic pumps for drug delivery. Plasma samples from animals with saline infused osmotic pumps had negligible levels of relaxin. After isolation of cardiac tissues from mice 28 days post MI, qPCR analysis was performed to quantify the expression of several pro-inflammatory and pro-fibrotic markers. The expression of pro-inflammatory (Irak3, Lta, Tlr2 & Tlr4), pro-apoptotic (Bax, Casp3), and pro-fibrotic genes (Tgfb, Lgals3, & Timp 1) was significantly lower in serelaxin-treated mice compared to control mice (Fig. 3).

### Treatment with serelaxin variably alters the expression of SphK enzymes

**Figure 4:**
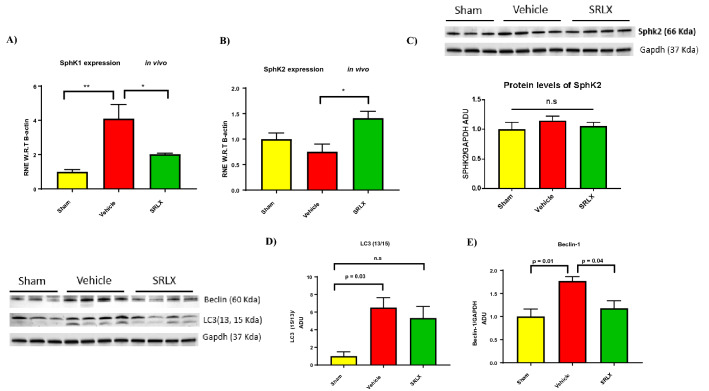
Expression of SphK1, SphK2 and autophagy markers in mice treated with serelaxin. (**A**,**B**) SphK1 gene expression is significantly lower in animals treated with serelaxin. Contrastingly, SphK2 gene expression is significantly higher in animals treated with serelaxin (**C**) SphK2 protein (70 kDa) levels are not significantly different between the vehicle treated and serelaxin treated groups For (**A**–**C**) (n = 4 for vehicle and SRLX groups), one-way ANOVA was utilized, followed by Tukey’s post hoc analysis. (**D**) The expression of the cleaved, active form of LC3 (13 KDa) is significantly higher in the vehicle group (when compared to sham mice). Contrastingly, no significant difference in LC3-13/15 ratio was observed between the sham and serelaxin treated groups; (**E**) The expression of the autophagy marker Beclin-1 (60 kDa) is significantly lower in mice treated with serelaxin 28 days post MI. For (**D**,**E**) n = 4 for vehicle and SRLX groups and n = 3 for sham group. One-way ANOVA with Tukey’s post-hoc analysis was utilized. Western blots for figures (**C**) and (**D**) were obtained from the same gel blot, and utilize the same GAPDH band (37 kDa) as can be seen in (**C**) and (**D**).

The in vitro experiments performed earlier show induction of SphK enzyme activity upon incubation with serelaxin. Subsequently, we tested whether the expression of the enzymes SphK1 and SphK2 were altered in diseased mice that received treatment. The mRNA expression of SphK1 is lowered upon treatment (Fig. 4A), but the expression of SphK2 mRNA is elevated in cardiac tissue samples from serelaxin-treated mice (Fig. 4B). However, when protein levels of SphK2 were tested, there were no significant changes among the Sham, Vehicle (saline) and Serelaxin treated groups (Fig. 4C).

### Long term treatment reduces the expression of autophagy markers

The role of autophagy in the pathophysiology of ischemic remodeling of the heart is yet to be fully elucidated, and its implications vary with the nature of injury (reperfused vs. non reperfused tissue) and the time course (acute vs. chronic stages of remodeling)^16^. We investigated whether relaxin signaling influenced the expression of mediators of autophagy in ischemic heart failure. During the initiation of autophagic processes within the cell, LC3-1 is cleaved and lipidated to generate LC3-II, which is recognized as a marker of autophagy^17,18^. Animals treated with saline had a stronger expression of the lipidated, cleaved form 28 days post MI, compared to the sham group (Fig. 4D). The ratio of LC3-II/LC3-1 did not vary significantly between the sham and serelaxin treatment groups. Beclin 1—a component of the PI3K complex that recruits Atg (Autophagy-related genes) proteins for the formation of the autophagosome^17,18^—is also elevated in saline treated animals (Fig. 4E).

### Relaxin signaling in ischemic heart failure and sphingolipid levels

**Figure 5:**
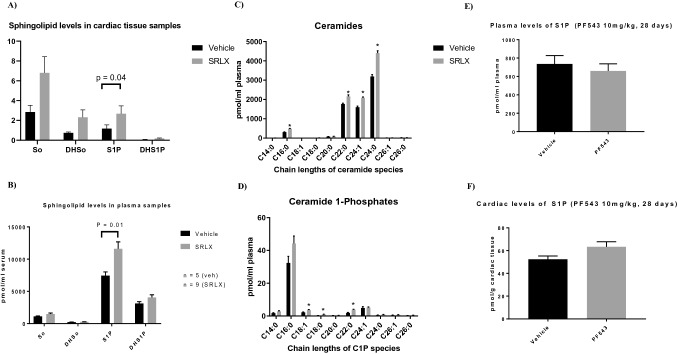
Lipidomic analysis of plasma and cardiac tissue samples from animals treated with serelaxin. (**A**,**B**) S1P levels are significantly higher in cardiac tissue and plasma samples extracted from animals receiving chronic serelaxin treatment. No significant differences were observed in sphingosine (So), dihydrosphingosine (DHSo), and dihydro S1P (DHS1P) levels in either cardiac tissue or plasma samples with treatment. For (**A**,**B**) unpaired T-test was performed within data sets of the same type. (**C**) Analysis of plasma ceramides (treated vs. untreated) shows significantly higher levels of C24:0 (*P* = 0.000006), C24:1 (*P* = 0.000008), C16:0 (*P* = 0.000011), and C22:0 (*P* = 0.0023) ceramides in extracts from serelaxin treated mice; (**D**) Plasma ceramide 1-Phosphate (C1P) levels are significantly higher in serelaxin treated mice, achieving significance in C22:0 (*P* < 0.0013), C18:1 (*P* = 0.0099) and C18:0 (*P* = 0.014) C1P levels. For A and B, n = 6 for vehicle, and n = 9 for serelaxin treated mice. For (**C**,**D**) unpaired T-test was used for comparisons. (**E**,**F**) Plasma and cardiac levels of S1P were not significantly lower with daily, i.p. injections of PF543 dissolved in the amphiphilic solvent Kolliphor HS 15.

Based on our earlier results suggesting increased SphK enzyme activity upon acute and sub-acute treatment with serelaxin, we investigated the cardiac and plasma levels of sphingolipids 28 days post MI, in both the saline and serelaxin treated groups. Flash frozen samples were submitted to the VCU lipidomics core for sphingolipid analysis. Lipids were extracted via the Bligh–Dyer method, followed by sample analysis via HPLC and linear ion trap mass spectrometry. Analysis of both plasma and tissue samples showed higher levels of S1P in the serelaxin treated group (Fig. 5A,B). While modest increases in sphingosine, dihydrosphingosine (DHSo) and dihydro S1P (DHS1P) were also noted, differences between the groups failed to reach statistical significance. Interestingly, a trend of increased ceramide levels in both plasma and tissue samples were observed upon treatment with serelaxin. The expression of long and very long chain ceramides (C24:0, C24:1, C16:0, C22:0) in plasma and the ultra-long chain ceramide (C26:1) was significantly elevated in the treated group (Fig. 5C). The expression of Ceramide-1 phosphates (C22:0, C18:1, C18:0) was also significantly higher in plasma samples from animals treated with serelaxin (Fig. 5D). Ceramide-1 phosphate levels in the cardiac tissue were not analyzed in this current study due to lack of sample availability.

### In vivo dosing of PF543 does not alter S1P levels significantly

Our results demonstrated reduced levels of autophagy in ischemic heart disease upon treatment with serelaxin. Given the correlation with elevated S1P levels (both cardiac and tissue concentrations), we attempted to inquire whether the upregulation of SphK activity was required for relaxin induced reduction in autophagy markers. Since the effect of serelaxin on S1P generation was primarily mediated through SphK1 activity in neonatal mouse cardiomyocytes^13^, we hypothesized that pharmacologic inhibition of SphK1 via PF543—a nanomolar inhibitor specific to regulating SphK1 activity^8^—would prevent serelaxin-associated increases in S1P, and attenuate the reduction in autophagy observed with treatment. In order to test the feasibility of using a primarily in vitro approach for in vivo usage, mice were injected with daily dosages (10 mg/kg) for 28 days, followed by lipidomic analysis of cardiac and plasma samples. PF543 was dissolved in Kolliphor (HS-15) due to its emulsifying properties. In vivo administration of PF543 failed to significantly reduce S1P levels in both tissue and plasma samples (Fig. 5E,F), necessitating investigation of alternative approaches for S1P reduction for further mechanistic insight.

## Discussion

Our model for long term serelaxin-induced protection from cardiac adverse remodeling recapitulates several of the findings demonstrated in previous studies incorporating a permanent ligation model for ischemic heart failure to examine the protective effects of treatment with relaxin. In Samuel et al.^10^, treatment with relaxin over a 30-day time period improved survival, reduced cardiomyocyte apoptosis and expression of TGF-β1 expression, curtailed pathologic myofibroblast differentiation and spurred angiogenesis^19^. Treatment with serelaxin also reduced cardiomyocyte apoptosis at 28 days as shown in Fig. 2C. We previously showed that reperfusion therapy with serelaxin reduces NLRP3 inflammasome activity, as demonstrated via reduction in caspase-1 activity. In this current study, in addition to confirming several of the findings demonstrated in Samuel et al.^10^, treatment with relaxin reduced the mRNA expression of galectin-3 (Lgals3) 28 days post MI in the cardiac tissue. This finding is supported by additional evidence from rats receiving serelaxin for 72 h post-acute MI^20^. Galectin-3 levels are inversely associated with LV reverse remodeling in clinical studies^21^ and pharmacologic inhibition of Galectin-3 reduced the extent of ischemia–reperfusion (IR) injury in mice, while mitigating cardiomyocyte apoptosis and mitochondrial dysfunction^22^. Therefore, the protective effects associated with the downregulation of galectin-3 further contribute to the pleiotropic effects of relaxin signaling in the ischemic heart.

In the current study, we report a novel finding involving the association of relaxin treatment and reduced induction of autophagy in the failing myocardium 28 days post MI. A potential link between relaxin signaling and autophagic signaling was previously demonstrated in vitro, where TGF β-induced fibrosis in cardiac fibroblasts led to increased autophagic flux, and relaxin reduced the lipidation of LC3B and p62 expression in a Stat-3 dependent manner^23^. Nevertheless, therapeutic targeting of autophagic processes in the ischemic myocardium requires further understanding of the role played by autophagy given the nature of the disease and the course of its evolution. In the normal heart, autophagy serves a vital role in eliminating dysfunctional organelles and damaged proteins (especially in the context of metabolic stress and nutrient deprivation), and suppressing inflammatory pathways from deviating cell fate toward apoptosis^17,18,24^. During acute myocardial ischemia, autophagy has been shown to confer cardioprotection, as deletions/mutations of proteins involved in the formation of the autolysosome (DRAM2, Ulk1, Atg7) either induce baseline cardiac dysfunction, or exacerbate MI pathophysiology. The usage of autophagy inhibitors such as bafilomycin A1, or disruption of the AMPK-mTOR pathway (due to its involvement in inducing autophagy during energy-starved states) lead to increased infarct size upon LAD ligation in animal models^17,18,24^. Contrastingly in animal models involving overt heart failure, excessive autophagy presents as an alternative modality of cardiomyocyte death and leads to further functional disruption^24^. In our study, mice receiving chronic serelaxin therapy demonstrated reduced expression of cleaved LC3B and beclin-1 expression. Given the current scope of this study, it is uncertain whether the influence of relaxin therapy led to a true decrease in autophagic markers, or whether the multifaceted treatment benefits associated with relaxin led to an overall improvement in protein and organelle health, reducing the demand for compensatory autophagic processes. Alternatively, animals from the vehicle (saline) group could have focal deficits in other steps associated with autophagy, and this could explain the increases in LC3B cleavage and beclin-1 in attempts to salvage the disrupted autophagic flux inside the remodeling myocardium. Furthermore, we analyzed total myocardial tissue for the expression of the aforementioned markers, but separation of the issue into peri-infarct and remote myocardium can lead to a more precise understanding of these mechanisms.

Chronic treatment with serelaxin led to increased levels of S1P in both plasma and tissue samples, along with associated increases in the upstream ceramide precursors. The interpretation of this result requires further study due to the complexity of the biochemical pathways involved in ceramide and sphingolipid generation and degradation. In Aragon-Herrera et al.^14^, increased expression of very long ceramides was also observed in rats treated with serelaxin. Ceramides are generally linked to pathologic pathways inside the diseased heart due to their induction of cellular apoptosis and senescence^25^. Increased ceramide content is especially associated with insulin resistance and lipotoxicity. De novo synthesis of ceramides has also been shown to increase in the failing myocardium obtained from patients with advanced heart failure^26^. However, the paradigm is further complicated by variable effects exerted by ceramides of different chain lengths. For instance, upregulation of the very long chain ceramide synthase (CerS2) increased oxidative stress, cell death and mitochondrial dysfunction in cardiomyocytes, but modulation of Cers5 (medium and long chain ceramide synthase) did not influence these pathways^27^. In contrast to the general paradigms associated with ceramide, ceramide 1 phosphates (C1P), which are synthesized through direct phosphorylation of ceramides via ceramide kinases, are known to exert pro-mitogenic mechanisms and activate cell survival cascades. In our study, C1P levels were upregulated in plasma samples from serelaxin-treated animals^25^. Whether these increases are downstream of increased ceramide flux in the cell, or are associated with direct activation or upregulation of ceramide kinases due to serelaxin-induced activity is yet to be elucidated. Additionally, investigation of the role of distinct ceramide kinases (Cers1-6), and the effects of relaxin signaling on their expression/activity is also an important subject for future studies.

Based on our results, relaxin induced S1P increases can probably be attributed to SphK activity levels. In Frati et al.^13^, the authors attribute increases in S1P levels upon relaxin treatment primarily to increases in SphK1, but not SphK2, activity. In our study, it was uncertain whether changes in SphK enzyme levels contributed to increased S1P levels. Treatment with relaxin led to reduced transcription of sphk1, and increased mRNA levels of sphk2 28 days post MI. However, protein levels of SphK2 were not significantly altered among the experimental group. The lack of a reliable antibody for SphK1 thwarted further analysis. Nevertheless, increases in S1P due to relaxin-induced stimulation of SphK activity has potent therapeutic considerations. SphK activity is declined in the remodeling heart post MI, and oral administration of S1PR1 agonist led to reduced apoptosis and preserved function 2 weeks post MI^28^. Adenoviral gene therapy involving S1P receptor delivery protected cardiac function through inhibition of beta-adrenergic activity (through receptor crosstalk) in a rodent model of ischemic heart failure^29^. However, manipulation of the S1P-S1PR axis in cardiac pathology is also dependent on the window of treatment, as S1PR1 agonists have been shown to induce lethal arrhythmias immediately after MI, while conferring protection when administered prior to or after the acute phase of MI^28^. In Zhang et al.^8^, usage of the S1P analog FTY720 (Fingolimod, which is now approved for therapy in multiple sclerosis), led to decline in membrane S1PR1 levels (through prolonged receptor activation and subsequent internalization) and subsequently protecting cardiac function, in contrast to the findings mentioned above. Usage of the SphK1 inhibitor PF543 (1 mg/kg, dissolved in 2% DMSO in saline and delivered i.p, for 28 days) in vivo also recapitulated similar results in the same study, implicating a disruption of the S1P-SphK1-S1PR1 axis as potentially cardioprotective^8^. In our study, PF543 formed an immiscible suspension at various dilutions in DMSO/saline. We chose Kolliphor (HS-15) due to its emulsifying properties as a vehicle for subsequent experimentation. However, even a higher dose of PF543 (10 mg/kg, dissolved in a HS-15 /saline solution) failed to reduce either plasma or cardiac tissue levels of S1P, in contrast to the findings in Zhang et al. where PF543 (1 mg/kg) reduced cardiac and plasma S1P levels. Further study is necessary to resolve the discrepancy in findings, but one possible explanation involves the difficulty associated with sample preparation prior to lipidomic analysis, since presence of blood due to inadequate cleaning of cardiac tissue post extraction could significantly influence results, as RBCs are known to contain high levels of S1P^1^. Due to the lack of SphK1 inhibition in vivo from PF543 in our study, we were unable to demonstrate whether the correlation between elevated S1P levels from relaxin therapy and reduced expression of autophagy markers 28 days post MI were linked. In Chen et al.^7^, treatment of cardiomyocytes directly with S1P reduced the number of autophagosomes in an in vitro model of phenylephrine and starvation-induced hypertrophy. Subsequently, knockdown of S1PR1 in vivo induced hypertrophy and stress-associated autophagy, along with impaired function^7^. Therefore, future studies might require alternative strategies for SphK1 inhibition for further mechanistic insight. Administration of myriocin (inhibitor of sphingosine biosynthesis) or usage of Sphk^−/−^ transgenic mice could also be considered for further experimentation. Alternatively, serelaxin might be associated with pathways involved in sphingolipid biodegradation, affecting bioavailability. The interactions of serelaxin with S1P lyase, the enzyme primarily involved with sphingolipid biodegradation, are also a subject of further investigation.

In summary, despite the limitations imposed by the lack of a working strategy for SphK1 inhibition and the elaborate nature of the signaling pathways described, our results show prevention of adverse remodeling in mice with ischemic heart failure upon treatment with serelaxin. In addition to inhibition of several pro-inflammatory and pro-fibrotic markers, we show a novel correlation between relaxin therapy and cardiac/plasma S1P levels in the context of ischemic heart disease. This correlation is supported by our data in primary cardiomyocytes, where relaxin increased the activity of SphK1 upon acute and subacute exposure. Therefore, the intersection of relaxin signaling and sphingolipid biology is a potential therapeutic target for cardiac health and disease.

## Methods

### Animals

All experiments in this study were performed in strict adherence to guidelines for the case and use of laboratory animals, as updated by the NIH (8th edition, 2011), and the current study is reported in adherence to ARRIVE guidelines. The animal techniques employed in this study are approved by the Virginia Commonwealth University (VCU) Institutional Animal Care and Use Committee (IACUC). The study utilized adult CD1 male mice, of 6–8 weeks of age, which were purchased from Charles River Laboratories (Wilmington, MA). The animals were allowed to acclimate in a temperature-controlled vivarium for up to a week, with ample access to water and food, prior to subsequent experimentation.

### Drugs and chemicals

Reagents used in cardiomyocyte isolation (NaCl, KCl, MgSO_4_, CaCl_2_, NaHCO_3_, 2,3-Butanedione Monoxime, glucose, protease XIV from streptomyces griseus and taurine), were purchased from Sigma-Aldrich (St. Louis, MO). The collagenase II component of the digestion buffer was purchased from Worthington Biochemical Corporation (Lakewood, NJ). D17 Sphingosine for the sphingosine kinase (SK) assay was purchased from Avanti Polar Lipids (Birmingham, AL), and phosphatase inhibitors and fatty acid free BSA were obtained from Fisher scientific. Recombinant relaxin (serelaxin) was provided by Novartis pharmaceuticals (Basel, Switzerland). PF543 was obtained from Cayman Chemicals (Ann Arbor, MI). Kolliphor (HS-15) was obtained from Sigma-Aldrich.

### D17 sphingosine assay

The enzymatic activity of sphingosine kinase enzymes was quantified via the D17 sphingosine assay. Since D17 sphingosine is not a naturally occurring sphingolipid, measuring the levels of D17-S1P (the end product of SphK1/SphK2 enzymatic activity on the D17 sphingosine substrate) estimates the bioactivity of the kinases when subjected to experimental conditions. Primary cardiomyocytes subjected to serelaxin or control conditions for either 30 min (acute) or 24 h (subacute) were treated with d17 sphingosine (10 μM) for 30 min. For the acute treatments, this treatment occurred at the onset of exposure to serelaxin, resulting in a total exposure time of 30 min. After the treatment period, media and cells were separately harvested and sent for lipidomics analysis. After quantification of d17 S1P levels, data was normalized to total phosphate content per sample.

### Isolation of primary cardiomyocytes

Our protocol for isolation of primary adult murine cardiomyocytes is well established, and detailed in prior publication^30^. Briefly, cardiac tissue was isolated and perfused with a Ca^2+^ free myocyte isolation buffer via Langendorff apparatus. Digestion of extracellular matrix was performed via a collagenase II and protease XIV infused buffer, followed by centrifugation of digested tissue to collect the myocyte pellet. Myocytes were allowed to reconstitute in calcium reintroduction buffers prior to plating in laminin coated dishes.

### Osmotic pump implantation and permanent ligation

For in vivo experimentation, mice were implanted with Alzet osmotic pumps (Cupertino, CA) for sustained delivery of vehicle (saline) or treatment (serelaxin, at 10 μg/kg/day). Prior to implantation, the pumps were injected with vehicle or treatment, and incubated in saline for up to 12 h to facilitate quicker onset of diffusion of drug upon surgical implantation. Subsequently, the pumps were subcutaneously implanted in anesthetized mice (pentobarbital i.p, 70 mg/kg). Appropriate sedation was confirmed based on the lack of responsiveness to toe-pinch. After the pump implantation, thoracotomy was performed after intubation and mechanical ventilation. After visualizing the LAD artery, a 7–0 silk ligature was tied directly around the artery and fastened. Occlusion was visually confirmed through observed pallor and dyskinesia of the affected portions of the left ventricle. Post-operative analgesics (buprenorphine SR LAB, 0.5 mg/kg, s.c) and antibiotic (gentamicin, 0.7 mg/kg) were administered, in accordance with our animal surgery protocol. Animals were continually monitored until post-operative day 28, and were humanely euthanized for subsequent experimental analysis. The surgeon was blinded to the treatment allocation (vehicle vs. serelaxin).

### Echocardiography

Our detailed protocol for 2D echocardiography, M-mode image acquisition and data analysis is mentioned in prior publication^30^.

### Western blotting

Our detailed protocol for western blotting is outlined in previous publications^9,30^. Briefly, the isolated hearts were collected and washed in PBS, followed by a rapid freeze in liquid nitrogen. Following pulverization, a small amount of approximately 20 mg of whole tissue was then homogenized, sonicated, and centrifuged at 10,000×*g* for 15 min at 4 °C in RIPA buffer, with protease and phosphatase inhibitor cocktail added accordingly (Halt, ThermoScientific). A total of 100 µg of proteins per sample was resolved by SDS-PAGE on 10% acrylamide gels, transferred onto a nitrocellulose membrane, and blocked with 5% nonfat dry milk in Tris-buffered saline. Before incubation with primary antibodies, the membranes were cut into different sections to allow the acquisition of the images at different exposure times while minimizing the need to use a western blot stripping buffer. The primary antibodies used in this study include Sphk2 (Cat no: 17096-1-AP) (1:1000) from Proteintech (Rosemont, IL), and Beclin-1 (#3738) (1:1000), LC3A/B (#4108) (1:1000), GAPDH (#2118) (1:2000) were obtained from Cell Signaling Technology (Danvers, MA). All membranes were then developed with enhanced chemiluminescence and digitally acquired on the Chemidoc XRS system (Biorad). Protein expression was determined by densitometric analysis using ImageJ (National Institutes of Health).

### qPCR

A detailed protocol is described in previous publication^30^. For quantifying the expression of multiple genes after the experimental period, the predesigned qPCR plate for myocardial infarction (PrimePCR) was purchased from BioRad (Hercules, CA). LV samples, including both the scar and the remote LV myocardium, were utilized for mRNA extraction for both experimental groups.

### TUNEL and trichrome blue staining

Tissue samples at the 28-day timepoint were processed, paraffinized, and sectioned as mentioned in our previous literature. For estimation of LV scar, trichrome staining was performed, and the scar size was estimated and expressed as a ratio of left ventricular cross-sectional area. Image analysis was performed using ImageJ software (NIH). Myocardial apoptosis was estimated at day 28 via TUNEL staining. The reagent kits for trichrome staining and TUNEL assay were purchased from ThermoFisher (Waltham, MA).

### Lipidomics analysis

In vivo and in vitro samples were sent to lipidomic analysis for estimation of sphingosine (So), Dihydrosphingosine (DHSo), S1p, DHS1P (Dihydrosphingosine 1-phosphate), and ceramide and ceramide phosphates (C1P) of various chain lengths. Sample preparation, extraction (Bligh-Dyer method), analysis and quantification of final results were performed by the VCU Lipidomics core. Services and products in support of the research project were generated by the VCU Massey Cancer Center Lipidomics Shared Resource, supported, in part, with funding from NIH-NCI Cancer Center Support Grant P30 CA016059.

### Statistical analysis

Data from all the experiments was analyzed for normality in distribution via the Shapiro–Wilk normality test. For normally distributed data, unpaired T-test was utilized for comparing two data sets. For three or more groups, ANOVA was utilized, with Tukey’s post-hoc testing for multiple comparisons within the groups. For non-normally distributed data, Wilcoxon Rank Sum test was used. The null hypothesis was rejected if the *P* value was lower than 0.05. Data analysis and statistical testing were performed on Graphpad Software Inc. (version 8).

## Supplementary Information

Supplementary Information.

## Data Availability

Raw experimental data have been provided in a supplementary spreadsheet.
